# Efficiency Analysis of New Rural Cooperative Medical System in China: Implications for the COVID-19 Era

**DOI:** 10.3389/fpsyg.2021.686954

**Published:** 2021-05-28

**Authors:** Ke Song, Wei-Bai Liu, Yan Qing, Meng-Nan Tian, Wen-Tsao Pan

**Affiliations:** ^1^School of Economics and Management, Hunan University of Science and Engineering, Yongzhou, China; ^2^School of Foreign Languages, Hunan University of Science and Engineering, Yongzhou, China

**Keywords:** two-stage network idea, DEA-Malmquist, New Rural Cooperative Medical System, COVID-19 pandemic, efficiency

## Abstract

The sudden outbreak of coronavirus disease 2019 (COVID-19) has caused a huge impact on the Chinese residents' health and economic level. In the pandemic background, the country and its institutions have introduced pandemic-related insurance to stabilize the national situation. At this stage, insurance has played an increasingly important role in social life. With the popularization of insurance, the idea of buying insurance to avoid risk has gradually become popular among people. Among them, the New Rural Cooperative Medical System (NRCMS) has been farmers' common choice. The NRCMS, a mutual aid system created by farmers spontaneously in the country, plays a great role in guaranteeing farmers access to basic health services, alleviating poverty caused by disease and returning to poverty due to disease, and promoting poverty alleviation and rural revitalization. Given this backdrop, we study the efficiency of the NRCMS that can effectively promote poverty alleviation and rural revitalization and ensure the people's happy life. Implementing the Data Envelopment Analysis (DEA), we find that technological progress is one of the main factors influencing the efficiency of the NRCMS. Therefore, it is important to improve the technology for providing the efficiency of the NRCMS and promoting the happiness of the society.

## Introduction

Coronavirus disease 2019 (COVID-19) continues to appear in many countries, which has brought many bad effects to the country and society. As of March 31, 2021, the number of infected people globally is almost 130 million, and the number of cases is increasing by more than 600,000 every day. The spread of infection affects all countries and regions worldwide and has caused more than 2.8 million deaths worldwide (Dong et al., 2020). This outbreak has infected a large number of people. The pandemic, which has infected so many people and spread so fast, is the most serious infectious disease disaster human society has experienced in nearly 100 years. Now, the global COVID-19 pandemic has not been effectively contained, and many European countries are beginning to experience a third-wave of the pandemic. However, if the global scientific and medical fields spare no effort to combat the pandemic, the future of the pandemic is uncertain. However, governments' policy and trust in these policies are central to combat the pandemic (Gozgor, 2021).

At this stage, we need to deeply analyze the impact of COVID-19 on the country and society, improve the long-term mechanism for global public security, improve the global governance system, and accelerate a community building with a shared future humankind. Under the pandemic situation, the New Rural Cooperative Medical System (NRCMS) has also played a certain role. The NRCMS was formally proposed by the Chinese government in October 2002 to actively guide farmers to establish a new type of rural cooperative medical insurance based on overall planning for serious diseases. In 2009, the NRCMS was established for the status of the rural basic medical security system. Until now, the NRCMS in the country has experienced a leap-forward development, reduced a lot of burden to farmers, and at the same time given a certain security. Many scholars study the effect of new farming in succession, and the corresponding research is as follows below.

For instance, Zhao and Gan (2019) proposed that the NRCMS improves the rate of rural residents receiving medical treatment, plays a significant role in reducing residents' medical expenditure, alleviates the acceptance pressure of municipal hospitals, guides rural residents to seek medical treatment nearby, and improves the utilization rate of medical services. Wang et al. (2009) proposed that participating in the NRCMS can effectively improve rural residents' medical services. Guo (2020) showed that under different risk preferences, farmers' participation in the NRCMS would also change and analyzed the influence of subjective and objective risk preferences on their insurance behavior. Guo et al. (2013) used the gray correlation analysis method to get the gray correlation order of the influencing factors of the NRCMS. The NRCMS should be designed and improved to adapt to objective conditions to achieve sustainable development. Cui et al. (2021) studied the province's livability level measurement based on the two-stage Data Envelopment Analysis (DEA) model. The authors comprehensively evaluated the livability level of the province from seven aspects. Xiao and Yang (2020) evaluated listed companies' innovation efficiency in China's wine industry based on a two-stage network DEA. They concluded that there were great differences in the innovation efficiency among listed companies in China's wine industry. There was an imbalance in the input and output of the technology development stage in innovation activities. Wang (2020) concluded that the new energy industry's overall financing efficiency was relatively low by constructing a two-stage chain network DEA model. Chen and Guan (2021) concluded that the allocation efficiency of health resources in traditional Chinese medicine (TCM) hospitals in China was poor using the DEA–Malmquist index. Jin et al. (2021) carried out static and dynamic analyses on the efficiency of inclusive digital finance regulating urban and rural residents' welfare differential in 31 provinces of China based on the DEA–Malmquist–Tobit model. The authors provided the main factors affecting inclusive digital finance's efficiency regulating urban and rural residents' welfare differential. Yang et al. (2021) studied county-level public hospitals' operational efficiency in Hainan Province based on the DEA–Malmquist model. The authors proposed the need to raise the technical efficiency and the scale efficiency.

In conclusion, the NRCMS has played an important role in recent years. Further exploration of the efficiency of the NRCMS can help better implement the NRCMS policies. In this paper, the two-stage network DEA and the DEA–Malmquist are used to study the efficiency of the NRCMS. There are few research studies on the NRCMS by this method in China, which helps explore the key influencing stages of the NRCMS to determine the important influencing factors. We enhance the previous papers by using new methods and provide some implications on the COVID-19 era.

The rest of the paper is organized as follows. The Methodology section explains the methodologies used in the paper. The Empirical Analysis section provides the details of the empirical analyses. The Conclusion section concludes.

## Methodology

### Two-Stage Network DEA

The specific model of the two-stage network DEA is constructed as follows: suppose there are many influencing factors, and each influencing factor has *n* inputs, *m* outputs, and *g* intermediate outputs, where Xi is the input of the first-stage fundraising system of the i-th influencing factor (DMU), Xi = (xi1, xi2..., xin)^T^; Yi is the output of the first-stage fundraising system and the input of the second-stage fund allocation system of the i-th decision unit, Yi = (Yi1, Yi2...Yig)^T^; Zi is the output of the fund allocation system in the second stage, ZI = (ZI1, ZI2... ZIN); and O = (O1, O2..., ON), P = (P1, WP2..., pg), Q = (q1, q2..., qm), respectively, represent the weights of input, intermediate, and output variables.

(1) $$\{\begin{array}{l} {\text{OTx}}_{0}=\text{1}\\ \text{QTYi}-{\text{O}}^{\text{T}}\text{Xi}\leq 0\\ \text{s}.\text{t}.\text{PTYi}-{\text{O}}^{\text{T}}\text{Xi}\leq 0\\ \text{QTZi}-{\text{P}}^{\text{T}}\text{Yi}\leq 0\\ \text{Q}\geq \varepsilon \text{em},\text{O}\geq \varepsilon \text{ep},\text{P}\geq \varepsilon \text{eg} \end{array}$$

Where ε is a non-Archimedes infinitesimal quantity, e T = (1, 1...1). If O^*^, P^*^, and Q^*^ are the optimal solution of the model, then the efficiency of DMU whole process and sub-process is E_0_ = Q^*^^T^Z_0_ O^*^^T^X_0_, E1 = Q^*^^T^Z_0_/O^*^^T^Y_0_, E2 = Q^*^^T^Z_0_/O^*^^T^Y_0_, where E_0_ is the whole stage, E1 and E2 are the first and second stages, respectively, and E_0_ = E1 × E2.

### DEA–Malmquist Method

Fare et al. (1994) combined a nonlinear programming method of the Malmquist index theory with the DEA method theory, making the Malmquist index widely applied in the empirical literature. This method has been widely applied to measure production efficiency in finance, industry, medical treatment, and other sectors, and international comparative studies have been conducted based on efficiency measurement results. The specific formula is as follows:

suppose (xt,yt) represents the input and output of the t period, (xt + 1,yt + 1) represents the input and output of the t + 1 period, and D_0_(xt + 1,yt + 1)/D_0_(xt,yt) represents the change of technical efficiency from t period to t + 1 period under the technical conditions of the t period; D_0_T + 1(X T + 1, Y T + 1)/D_0_T + 1(X T, Y T)]^1/2^ represents the change in technical efficiency from T to T + 1 under the technical conditions of T + 1, where the subscript 0 represents the constant return to scale. The following formula can express the Malmquist index:

(2) $$\begin{array}{l} {\text{M}}_{\text{0}}\left({\text{x}}^{\text{t}},{\text{y}}^{\text{t}},{\text{x}}^{\text{t+1}},{\text{y}}^{\text{t+1}}\right)\text{=}[{\text{D}}_{\text{0}}\left({\text{x}}^{\text{t+1}},{\text{y}}^{\text{t+1}}\right){\text{/D}}_{\text{0}}({\text{x}}^{\text{t}},{\text{y}}^{\text{t}})\\ {\text{×}{\text{D}}_{\text{0}}^{\text{t+1}}\left({\text{x}}^{\text{t+1}},{\text{y}}^{\text{t+1}}\right){\text{/D}}_{\text{0}}{\;}^{\text{t+1}}\left({\text{x}}^{\text{t}},{\text{y}}^{\text{t}}\right)]}^{\text{1/2}} \end{array}$$

At the same time, the Malmquist approach can be divided into two parts: technical efficiency and change. The specific formula is as follows:

(3a) $$\begin{array}{l} \text{Technical efficiency EC }={\text{D}}_{0}\text{T}+\text{ 1}\left({\text{X}}_{0}\text{T}+\text{ 1},{\text{Y}}_{0}\text{T}+\text{ 1}\right)\\ /{\text{D}}_{0}\left({\text{X}}_{0}\text{T},{\text{Y}}_{0}\text{T}\right) \end{array}$$

(3b) $$\begin{array}{l} \text{Technical change Tc }=\;\left[{\text{D}}_{0}\text{t}({\text{x}}_{0}\text{t}+\text{ 1},{\text{y}}_{0}\text{t}+\text{ 1}\right)\\ /{\text{D}}_{0}\left({\text{x}}_{0}\text{t}+\text{ 1},{\text{y}}_{0}\text{t}+\text{ 1}\right)\times {\text{D}}_{0}\text{t}+\text{ 1}\left({\text{x}}_{0}\text{t}+\text{ 1},{\text{y}}_{0}\text{t}+\text{ 1}\right)\\ /{\text{D}}_{0}\text{t}+\text{ 1}\left({\text{x}}_{0}\text{t},{\text{y}}_{0}\text{t}\right)] \end{array}$$

(3c) M = EC*TC

EC mainly refers to the utilization of existing technology by decision-making units if EC > 1 means that the efficiency of the main body of production is improved, resources are fully utilized, and management level is improved. On the contrary, if EC < 1, resources are not fully utilized, main body efficiency is poor, and management level needs to be strengthened. TC mainly reflects the technological progress on the decision-making unit if Tc > 1 indicates technological progress. Tc < 1 indicates no technological progress or regression, and continuous improvement of the technology is needed. Tc = 1 indicates no technological progress.

## Empirical Analysis

### Sample Description

In this paper, the data are selected from the year between 2004 and 2014, the ×1 [the new rural cooperative medical county (area and city)], ×2 (to participate in the new rural cooperative medical number), ×3 (the new rural cooperative medical service rate), ×4 (the new rural cooperative medical fund spending), ×5 (the total amount of the new rural cooperative medical financing this year), and ×6 (the new rural cooperative medical compensation benefit passengers). The data are obtained from the Chinese Longitudinal Healthy Longevity Survey (CLHLS) of Zeng et al. (2017). The descriptive statistics of raw dataset is reported in Table 1.

**Table 1:** Descriptive statistics of raw dataset.

**Time**	**X1** ** (PCS)**	**X2** ** (million people)**	**X3** ** (%)**	**X4** ** (million yuan)**	**X5** ** (million yuan)**	**X6** ** (million people)**
2014	2,349	7.36	98.9	2,890.4	3,025.28	16.52
2013	2,489	8.02	99	2,908	2,972.48	19.42
2012	2,566	8.05	98.3	2,408	2,484.7	17.45
2011	2,637	8.32	97.5	1,710.19	2,047.56	13.15
2010	2,678	8.36	96	1,187.8	1,308.33	10.87
2009	2,716	8.33	94.2	922.9	944.4	7.59
2008	2,729	8.15	91.5	662.3	784.58	5.85
2007	2,451	7.26	86.2	346.63	427.97	4.53
2006	1,451	4.1	80.7	155.8	213.59	2.72
2005	678	1.79	75.7	61.75	75.35	1.22
2004	333	0.8	75.2	26.37	40.29	0.76

*The input/output/intermediate variables of the two-stage network DEA are provided in Table 2. The input/output variables of DEA–Malmquist are also reported in Table 2*.

**Table 2:** Variables in the two-stage network DEA.

**Input/output**	**Names of index**	**Index tag**	**Units**
Input	Counties (districts and county-level cities) carrying out the new rural cooperative medical care system	X1	Pcs
	Number of people participating in the new rural cooperative medical care system	X2	Million people
	Expenditures for the new rural cooperative medical care system	X4	Million yuan
	New rural cooperative medical system this year total financing	X5	Million yuan
Intermediate	Participation rate of the new rural cooperative medical care system	X3	%
Output	The number of beneficiaries of the new rural cooperative medical care system	X6	Million people
**Variables in the DEA–Malmquist Method**			
Input	Counties (districts and county-level cities) carrying out the new rural cooperative medical care system	X1	Pcs
	Number of people participating in the new rural cooperative medical care system	X2	Million people
	Expenditures for the new rural cooperative medical care system	X4	Million yuan
	New rural cooperative medical system this year total financing	X5	Million yuan
Output	Participation rate of the new rural cooperative medical care system	X3	%
	The number of beneficiaries of the new rural cooperative medical care system	X6	Million people

### Results of the Two-Stage Network DEA Method

The results of the two-stage network DEA method are provided in Table 3.

**Table 3:** Results of the two-stage network DEA method.

**No**	**Node**	**DMU**	**Score**	**Ranking**
1	1	2004	1	1
2	1	2005	0.494416	13
3	1	2006	0.246282	20
4	1	2007	0.242821	21
5	1	2008	0.281633	17
6	1	2009	0.235476	22
7	1	2010	0.342022	15
8	1	2011	0.371864	14
9	1	2012	0.265954	19
10	1	2013	0.305135	16
11	1	2014	0.27504	18
12	2	2004	1	1
13	2	2005	1	1
14	2	2006	1	1
15	2	2007	0.831183	6
16	2	2008	0.585505	12
17	2	2009	0.759278	9
18	2	2010	0.784924	8
19	2	2011	0.642427	11
20	2	2012	0.845297	5
21	2	2013	0.786354	7
22	2	2014	0.657252	10

It can be seen from Table 3 that the overall efficiency of the second stage is higher than that of the first stage; there are 3 years with an efficiency value of 1 in the second stage, and only one year with an efficiency value of 1 in the first stage; the efficiency value of the second stage from 2009 to 2014 is generally high. Therefore, the first stage mainly affects the overall efficiency; in the year 2005, 2006, 2008, and other years, the efficiency of the first stage is poor, but the efficiency of the second stage is efficient, indicating that there may be technological progress in the second stage compared with the first stage.

### Results of the DEA–Malmquist Method

The results of the DEA–Malmquist method are provided in Table 4.

**Table 4:** Results of the DEA–Malmquist method.

**Time**	**Stage**	**EC**	**TC**	**PEC**	**SEC**	**Number of M**	**Ranking**
2005	A	1	0.607443	1	1	0.607443	9
2006	A	1	0.62593	1	1	0.62593	8
2007	A	1	0.688009	1	1	0.688009	7
2008	A	1	0.802715	1	1	0.802715	6
2009	A	1	0.981397	1	1	0.981397	2
2010	B	1	0.892905	1	1	0.892905	5
2011	B	1	0.990985	1	1	0.990985	1
2012	B	1	0.97817	1	1	0.978171	3
2013	B	1	0.953863	1	1	0.953863	4

As shown in Table 4, every year, the EC, PEC, and SEC's efficiency values are obtained as one. This evidence indicates an efficient situation. In addition, the efficiency value of the TC was not up to 1 every year. This case is a situation where there is no progress or deterioration in the technology. Still, with the increase of time, the efficiency value of the TC closer to 1 shows that the technology is improving year by year, but the efficiency value is no more than 1. Therefore, the most important reason why the *M-*value is <1 is that the TC value is <1. In terms of the ranking of the *M-*value, the top 5 years were 2010–2014, among which 2011 ranked first and 2010 ranked as the fifth.

## Conclusion

Our empirical analyses in this paper show that the first stage's efficiency is poor, the second stage's efficiency is efficient, and the overall efficiency is poor. Therefore, the first stage is the stage that mainly affects the overall efficiency. We suggest that the second stage is more efficient than the first stage for the following reasons. In 2009, the new agricultural cooperation was established as the basic medical security in rural areas. Then, from 2010 to 2014, relevant documents and policies were issued every year. For example, in 2010, the Eleventh Five-Year Plan Conference was held, requiring the NRCMS to cover more than 80% of rural areas by 2010. In 2011, the medical and health system's five key reforms were promulgated, which raised the standard of medical insurance subsidy from 120 to 200 RMB per person. Since then, the cost of medical insurance subsidies has been increasing continuously every year. In 2014, the Notice on Raising the 2014 Funding Standards for the New Rural Cooperative Medical Care and the Basic Medical Insurance for Urban Residents was issued. The subsidy for medical insurance was raised to 320 RMB per person, increasing 40 RMB than the subsidy in 2013. These subsidies are significantly increasing in the COVID-19 era, and it enhances the happiness and life satisfaction of the Chinese farmers. However, China's medical insurance funding level, especially basic medical insurance for rural and non-working urban residents, is still low. For example, vaccines are not included in the medical insurance coverage, and public health funds paid vaccinations.

To sum up, due to the implementation of relevant policies and each subsidy fee adjustment that is more in line with the national conditions, the NRCMS gradually develops well. In 2008, the overall efficiency and the first-stage efficiency were poor, but the second stage efficiency is better. This evidence means that the second stage is a technological improvement over the first stage. Technological progress is the main reason that affects the *M*-value, which is related to some less mature technologies of the NRCMS.

There are several implications, especially for the COVID-19 era. First, the procedures for participating in and applying for reimbursement for the NRCMS are tedious, and farmers are worried that the policy will change every year and not get adequate protection. Second, unable to cross area to hand in, new agriculture closes to want to attend domicile ground, cannot be in residence place to hand in, and protect personnel from ginning to bring certain not convenient. Third, it is inconvenient to reimburse other places, and the payment channel is single. It can only be reimbursed in the place of residence, and the round-trip fare is not necessarily cost-effective. Fourth, with the growth of years, technology keeps developing, but the efficiency value has not exceeded. Therefore, technology needs to be further improved, such as developing corresponding software to simplify the cumbersome insurance reimbursement procedures. In this software, one can see the payment cost and time of different places. One can also make payment and settlement in the corresponding software to get the corresponding guarantee regardless of where you live or in other places. In this way, the NRCMS will develop better and better and better guarantee farmers out of poverty and promote rural revitalization. The outbreak of the pandemic has also aroused people's deep thinking about rural medical treatment. The pandemic prevention and control in rural areas face great challenges because of the relatively weak medical and health conditions, the large number of people going out and returning home, and the large mobility. It is necessary to implement relevant policies of the NRCMS by combining the pandemic situation so that farmers can have insurance to rely on, and the NRCMS can play an important role during the pandemic period to strengthen the prevention and control in rural areas.

## Data Availability Statement

Publicly available datasets were analyzed in this study. This data can be found here: The data are obtained from the Chinese Longitudinal Healthy Longevity Survey (CLHLS) of Zeng et al. (2017): https://www.icpsr.umich.edu/web/ICPSR/studies/36692.

## Author Contributions

KS: implementing empirical analyses. W-BL: writing the manuscript. YQ: data collection and methodology. M-NT: reviewing the manuscript. W-TP: funding access. All authors contributed to the article and approved the submitted version.

## Conflict of Interest

The authors declare that the research was conducted in the absence of any commercial or financial relationships that could be construed as a potential conflict of interest.
